# *Omi*, a recessive mutation on chromosome 10, is a novel allele of *Ostm1*

**DOI:** 10.1007/s00335-012-9438-7

**Published:** 2012-11-17

**Authors:** Erika A. Bosman, Jeanne Estabel, Ozama Ismail, Christine Podrini, Jacqueline K. White, Karen P. Steel

**Affiliations:** Wellcome Trust Sanger Institute, Wellcome Trust Genome Campus, Hinxton, Cambridge, CB10 1SA UK

## Abstract

Large-scale *N*-ethyl-*N*-nitrosourea (ENU) mutagenesis has provided many rodent models for human disease. Here we describe the initial characterization and mapping of a recessive mutation that leads to degeneration of the incisors, failure of molars to erupt, a grey coat colour, and mild osteopetrosis. We mapped the *omi* mutation to chromosome 10 between *D10Mit214* and *D10Mit194*. The *Ostm1* gene is a likely candidate gene in this region and the *grey-lethal* allele, *Ostm1* ^*gl*^, and *omi* mutations fail to complement each other. We show that *om/om* mice have reduced levels of Ostm1 protein. To date we have not been able to identify the causative mutation. We propose that *omi* is a novel hypomorphic mutation affecting *Ostm1* expression, potentially in a regulatory element.

**Electronic supplementary material:**

The online version of this article (doi:10.1007/s00335-012-9438-7) contains supplementary material, which is available to authorized users.

## Introduction

Large-scale *N*-ethyl-*N*-nitrosourea (ENU) mutagenesis has provided many rodent models for human disease, including several forms of deafness, Branchio-Oto-Renal syndrome, and CHARGE syndrome (Hrabe de Angelis et al. 2000; Smits et al. 2005; Bosman et al. 2005, 2009; Calvert et al. 2011; Hilton et al. 2011). Due to the nature of production of these mutant mice, it is likely that additional mutations are present in the mouse lines studied because mutations are generated at random in the genome and are usually noticed only when they lead to a detectable phenotype. Some mutations may be without any obvious consequence, but other mutations may have additional phenotypes of varying degree that may go undetected because they are not screened for or they may appear in later generations if they are recessive. It has been estimated that an average of around 150 mutations occur in each F1 offspring from an ENU-mutagenized male (Hrabe de Angelis et al. 2000). Many of these additional mutations will be lost by dilution in successive generations through crossing to the wild-type background (Hilton et al. 2011) or by selection against the mutant phenotype; however, some will remain.

We report here a new recessive phenotype that was found in a line selected for a completely different phenotype originating from an ENU mutagenesis screen. The phenotype was initially detected due to high lethality just after weaning in some matings within this mouse line. Here we describe the initial characterisation and mapping of this mutation, that we named *omi* (*om*). *Omi* is a recessive mutation that leads to degeneration of the incisors, failure of molars to erupt, a grey coat colour, and mild osteopetrosis. We mapped the *omi* mutation to chromosome 10 between *D10Mit214* and *D10Mit194*. The *Ostm1* gene is a likely candidate gene in this region, and the *grey-lethal* allele, *Ostm1* ^*gl*^, and *omi* mutations fail to complement each other. We show that *om/om* mice have reduced levels of Ostm1 protein. To date we have not been able to identify the causative mutation. We propose that *omi* is a novel hypomorphic mutation affecting *Ostm1* expression, potentially in a regulatory element.

## Materials and methods

### Mice

Animal husbandry and experiments were carried out in accordance with UK Home Office regulations. The *omi* mutation was detected in a line of mice derived from a large-scale mutagenesis screen (Hrabe de Angelis et al. 2000). Male C3HeB/FeJ mice were injected with three doses of 80 mg/kg ENU at weekly intervals, allowed to recover, and mated with uninjected C3HeB/FeJ females. F1 offspring were screened for a variety of dominantly inherited defects, including deafness and vestibular (balance) defects. The recessive *omi* mutation was discovered in a mouse line initially isolated because of its dominantly inherited mild head-bobbing behaviour (ABE9, also known as Bob). We were unable to map the original feature of mild head bobbing to any chromosome and no obvious malformation of the inner ear could be detected (Bosman and Steel, unpublished results). Offspring from the original ENU-mutagenized male were outcrossed to wild-type C3HeB/FeJ mice (never exposed to ENU) at least five times, diluting out other mutations resulting from the ENU treatment, before the *omi* phenotype was discovered. The *omi* phenotype was not linked to the head bobbing, and mice described here showed no sign of any balance defect. Affected *omi* mice were provided with a Pico-Vac^®^ soft dietary supplement (LabDiet) which enabled them to survive until adulthood. For all experiments control animals were fed normal diet pellets *ad libitum*. For all phenotypic analyses the mice were studied on their original C3HeB/FeJ genetic background and unaffected wild-type or heterozygous littermates were used as controls. *Grey-lethal* mice were obtained from Prof. T. Jentsch and were genotyped as described previously (Chalhoub et al. 2003).

### Genetic mapping

*Om*/*om* animals on a C3HeB/FeJ genetic background were outcrossed to C57BL/6J mice. +/*om* offspring were backcrossed to affected (*om*/*om*) C3HeB/FeJ animals. Backcross mice were collected around weaning time. The mice were killed by cervical dislocation, the teeth were photographed, and tissues were taken for DNA purification. The DNA from 28 backcross offspring exhibiting tooth defects was used in a genome scan to link the tooth defect with a chromosome. A panel of 69 markers spanning the autosomes was used to detect polymorphisms between C3HeB/FeJ and C57BL/6J mice (Supplementary Table 1). For further fine mapping, another ten polymorphic markers were used (Supplementary Table 2). Single nucleotide polymorphisms (SNPs) between C57BL/6J and C3HeB/FeJ in the region of interest were identified using a SNP database (http://www.informatics.jax.org/javawi2/servlet/WIFetch?page=snpQF). Primers spanning the SNPs were designed using Primer3 (http://frodo.wi.mit.edu/) with standard settings (Supplementary Table 2 for primer sequence). PCR products were then sequenced with the forward and reverse primers to detect the SNP using standard techniques.

### Phenotypic analysis

Adult mice were killed by cervical dislocation. For histology, organs of 4-week-old male and female mice (*n* = 10; five *om/om* and five +/*om*) were collected and fixed in 10 % neutral buffered formalin. Heads were subsequently decalcified in 10 % EDTA (pH 8.0) for 7 days, followed by paraffin embedding. Embryos (E16.5, *n* = 13 from heterozygous × homozygous timed matings) were dissected in ice-cold PBS and decapitated, and the heads were fixed in 10 % neutral buffered formalin for 2 days and processed for paraffin embedding. P7 pups (*n* = 10 from heterozygous × homozygous matings) were decapitated and the heads were fixed in 10 % neutral buffered formalin and decalcified in 10 % EDTA (pH 8.0) for 7 days, followed by paraffin embedding. Serial sections at 7 μm were cut and stained with haematoxylin and eosin by standard procedures. To analyse the skeletons, mice (seven male +/*om*, four male *om*/*om*, seven female +/*om*, and four female *om*/*om*) were weighed and anaesthetised by intraperitoneal injection of either Avertin (1.25 %) or 100 mg/kg ketamine/10 mg/kg xylazine (dosage of 0.1 ml/10 g mouse weight) at 6–8 and at 12 weeks of age. When ketamine/xylazine was used, the anaesthesia was reversed using a solution of 1 mg/kg Antisedan (0.1 ml/10 g mouse weight). Mice were scanned in a MX20 Specimen Radiography System (Faxitron X-Ray Corporation, Lincolnshire, IL, USA) in combination with Faxitron SR v1.2 software. At 12 weeks the density of the tissues was measured using a PIXImus Densitometer in combination with Lunar PIXImus 2 2.1 software. At 16 weeks of age, all nonfasted mice used for the radiographic skeletal analysis were subjected to a terminal retro-orbital bleed under anaesthesia induced by 100 mg/kg ketamine/10 mg/kg xylazine (dosage of 0.1 ml/10 g mouse weight). A complete blood count and clinical chemistry panel were analysed (http://www.sanger.ac.uk/mouseportal/phenotyping/MAHN/plasma-chemistry/). Whole blood was analysed with an automatic haematology analyser which uses spectrophotometry and volume impedance principle to measure white and red blood cell counts, mean corpuscular volume, haemoglobin, erythrocyte indices (haematocrit, mean corpuscular haemoglobin, mean corpuscular haemoglobin concentration, red blood cell distribution width), platelet counts, and mean platelet volume (scilVet Animal Blood Counter, RAB 015 A Ind.E, 22.02.01). Plasma chemistry was analysed using an Olympus AU400 for the following parameters: sodium, potassium, chloride, glucose, triglycerides, cholesterol, high-density lipoprotein, low-density lipoprotein (LDL), NEFAC, glycerol, amylase, alanine aminotransferase, alkaline phosphatase, creatine kinase, aspartate aminotransferase, total bilirubin, total protein, albumin, creatinine, urea, calcium, magnesium, iron, phosphate, lactate dehydrogenase, and uric acid.

### Western blotting

Adult mice (*n* = 2 per genotype) were killed by cervical dislocation and adult brains were frozen in liquid nitrogen and stored at −80 °C until further use. Brain tissue (7 ml per 0.379 g tissue) was lysed by homogenization in an ice-cold Dounce homogenizer in ice-cold DOC buffer [1 % sodium deoxycholate in 50 mM Tris-HCl (pH 9.0)] containing Complete protease inhibitors (Roche Applied Science, Indianapolis, IN, USA) according to the manufacturer’s instructions. The lysate was incubated in ice for 1 h, spun at 4,000 rpm for 1 h, and then cleared through a 5-μm filter. Protein concentration was determined and similar quantities were loaded on a 4–12 % acrylamide gel (Bio-Rad, Hercules, CA, USA) and blotted. Western blots were blocked for 4 h at 4 °C in TBS + 0.1 % Tween-20 (TBST) containing 5 % low-fat milk powder, rinsed in TBST, and then incubated in anti-OSTM1 (catalog No. HPA010851 from Atlas Antibodies, Stockholm, Sweden) diluted 1:2,000 in TBST + 3 % low fat milk overnight at 4 °C. Blots were rinsed and washed 3 × 10 min with TBST at room temperature, then incubated with anti-rabbit-HRP (Horse radish peroxidase) diluted 1:50,000 in TBST + 3 % low fat milk overnight at 4 °C. Blots were rinsed and washed 3 × 10 min and 2 × 30 min in TBST. An ECL Advance kit (Amersham GE Healthcare Life Sciences, Piscataway, NJ, USA) was used to detect HRP activity. Blots were then stripped, blocked, and incubated with anti-PSD95 (catalog No. MA1-046 from Affinity Biosciences, Burwood, VIC, Australia) as described above, except for the use of anti-mouse-HRP as a secondary antibody.

## Results

### *Omi* is a recessive mutation affecting teeth and coat colour

**Fig. 1:**
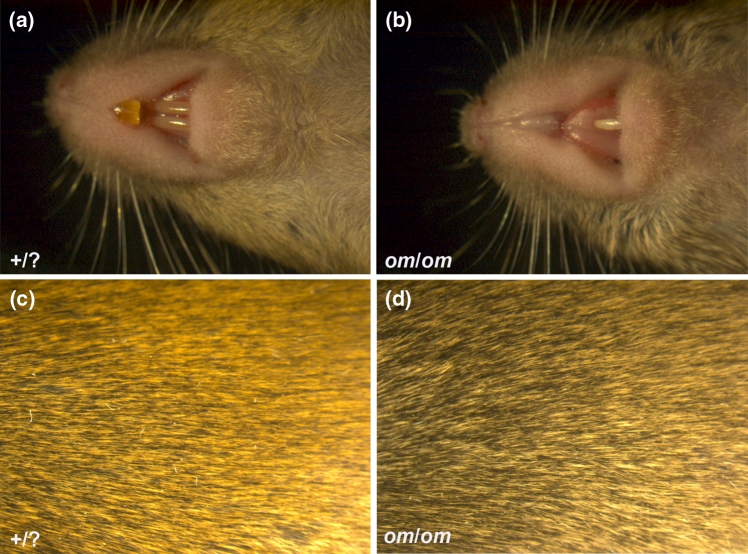
Coat colour and teeth of normal (+/+ or +/*om*) and *omi* (*om*/*om*) adult mice. **a** Normal mice have two upper and two lower incisors, **b** *om*/*om* mice lack most incisors. Any remaining incisors need clipping to prevent overgrowth, **c** A normal agouti coat colour in mice, **d** *om*/*om* mice have a less *yellow* coat

The recessive *omi* founders were discovered in a mouse line initially discovered because of dominant mild headshaking behaviour (ABE9), although the balance defect was not linked to the tooth defect. In a subset of matings of healthy parents, very small animals were found around the time of weaning, some of which died or had to be culled for welfare reasons. Providing these small mice with a special diet of soft food improved their survival rate. Analysis of these affected mice and normal littermates showed that these small mice lacked most incisors (Fig. 1a, b). In addition, we found that affected mice had a more grey (or less yellow) coat colour than their normal littermates (Fig. 1c, d).

We analysed the breeding records to establish inheritance. Of matings between healthy, unaffected individuals, we obtained either normal offspring only or a mixture of affected and normal offspring (Table 1). In the latter case, we found a ratio of affected:unaffected of approximately 1:3, indicating recessive inheritance. We crossed affected animals with normal animals from the colony and found that the offspring could be either all normal or a mixture of affected (41.4 %) and normal (58.6 %) (Table 1). Next we crossed an affected male with an affected female and we found that all ten offspring were affected, supporting the recessive inheritance.

**Table 1:** The *omi* mutation is a recessive mutation: numbers of normal and affected mice were analysed to determine mode of inheritance

Cross predicted genotypes	Unaffected × unaffected +/*om* × +/*om*		Unaffected × unaffected +/? × +/+		Affected × unaffected *om/om* × +/*om*		Affected × unaffected *om/om* × +/+		Affected × affected *om/om* × *om/om*	
Offspring	*n* = 110	(%)	*n* = 121	(%)	*n* = 162	(%)	*n* = 44	(%)	*n* = 10	(%)
Affected	25	22.7	0	0	67	41.4	0	0	10	100
Unaffected	85	77.3	121	100	95	58.6	44	100	0	0

When breeding these mice we noticed that some affected (*om*/*om*) mice appeared to have a shortened life span. In crosses where normal, unaffected males were used, these were in good health but culled due to a decrease in breeding performance around 7 months (*n* = 10; Table 2). However, seven *om*/*om* males were found dead and another 18 had to be culled because of ill health by 6 months (24 %; total *n* = 104). Similar lethality was found for *om/om* females (16 %; 13 of 79 females). Necropsy on these mice did not identify any obvious malformations. However, some nonbreeding *om*/*om* males (*n* = 2) survived to at least 1 year of age.

**Table 2:** *om*/*om* mice have a reduced lifespan

		+/?			*om*/*om*		
		FD	CS	CXS	FD	CS	CXS
Males	*n*	0	0	10	5	4	10
	average age (months)			7.2	6.0	5.6	6.7
	SD			1.9	1.4	1.7	1.9
Females	*n*	0	0	19	0	0	4
	average age (months)			7.8			6.3
	SD			3.0			2.8

*FD* found dead, *CS* culled sick, *CXS* culled excess

### Teeth are formed in *om*/*om* mice but are abnormal

**Fig. 2:**
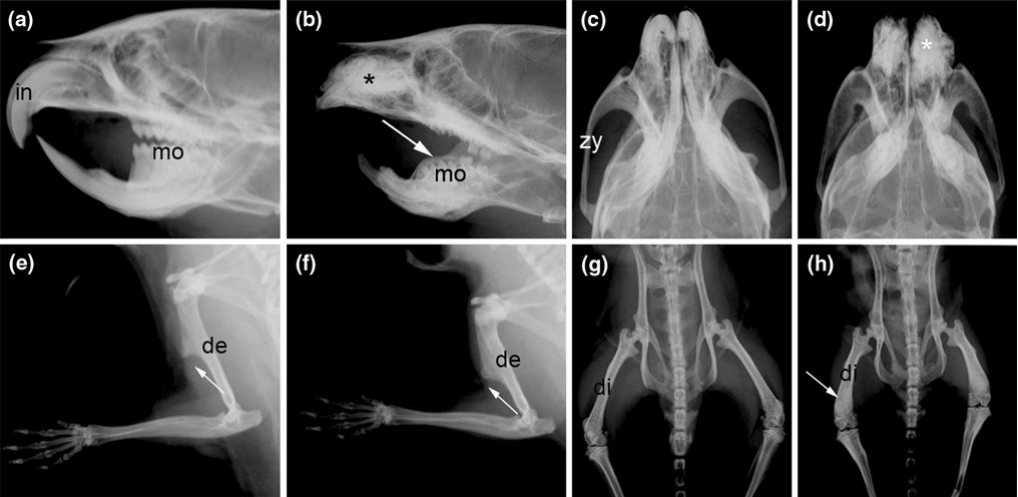
Analysis of tooth and bone abnormalities in normal (+/*om*) and *om/om* adult female mice. **a, b** Lateral view of the skull of 12-week-old normal (+/*om*) and omi (*om/om*) mice. In normal mice, the incisors (*in*) are clearly visible, and well-defined molars (*mo*) are visible in the oral cavity. In *om*/*om* mice, the incisors are reduced to stumps and misshapen molars appear covered by a tissue layer (*arrow*). In addition, fibrotic tissue is visible in the maxillary bone (*star*), **c, d** Dorsal view of the skull of 12-week-old normal (+/*om*) and *omi* (*om*/*om*) mice. Compared to normal mice, the zygomatic bone (*zy*) of *omi* mice is thickened. Fibrotic tissue is visible in the maxillary bone (*star*), **e, f** Lateral view of the front leg of 12-week-old normal (+/*om*) and *omi* (*om/om*) mice. The deltoid tuberosity (*de*) and deltoid process (*arrow*) are depicted. Note the difference in morphology of the deltoid process between normal and *omi* mice (*arrow*), **g, h** Dorsal view of the hind leg of 12-week-old normal (+/*om*) and *omi* (*om/om*) mice. In *omi* mice femurs are shorter but thicker and have increased density at the diaphysis (*di*) and the distal epiphysis (*arrow*)

To analyse the tooth defect and other skeletal problems over time, we took X rays at 6–8 and 12 weeks of age of +/*om* and *om*/*om* mice (Fig. 2). +/*om* males and females had normal incisors and molars at 6–8 and 12 weeks (Fig. 2a, data not shown). However, the morphology of incisors and molars was very abnormal in *om*/*om* mice (Fig. 2b). The incisors appeared short and the molars were not regularly aligned as in control mice. In addition, it appeared that some molars from *om/om* mice were still covered by other tissue indicating that these do not erupt (Fig. 2b). Sometimes upper molar 3 was visible within the buccal cavity but appeared damaged.

**Fig. 3:**
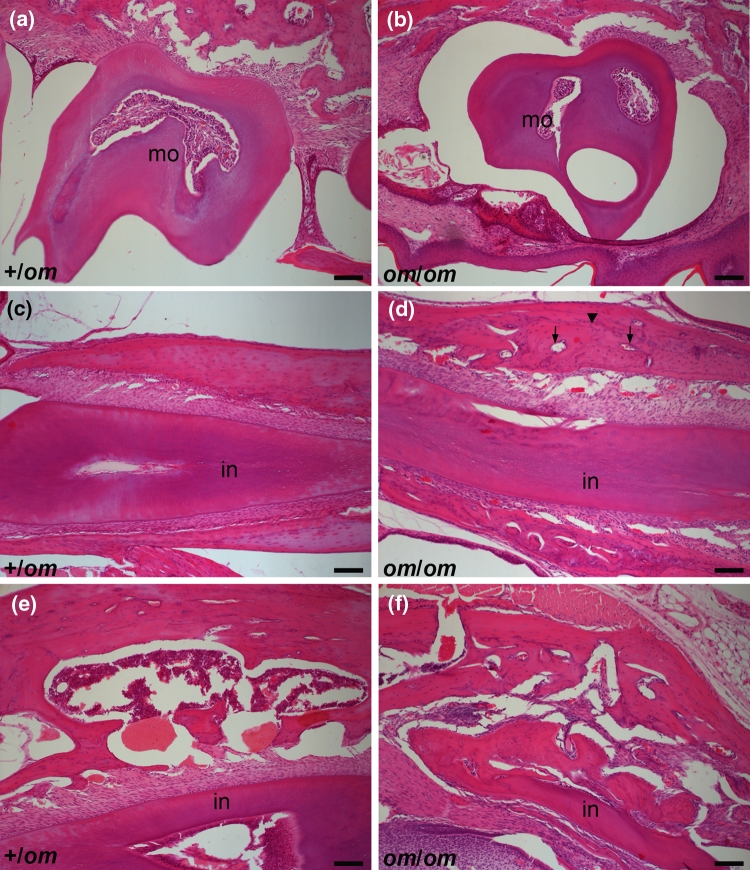
Analysis of tooth and bone abnormalities by histology in 3-week-old normal (+/*om*) and *om/om* mice. **a, b** Sections through molars of normal (+/*om*) and *omi* (*om/om*) mice. In normal mice molars (*mo*) have erupted and are located in the buccal cavity, whereas the molars of *om/om* mice remain in the jaw under several tissue layers, **c, d** Sections through incisors (*in*) of normal and *om/om* mice. The maxillary bone of normal mice has a compacted and regular structure (compacted bone, *cb*) with no blood vessels. In *om/om* mice the bone is disorganized (*arrowhead*) and has many blood vessels penetrating (*arrow*). *Scale bar* 100 μm

We sectioned heads of 3-week-old +/*om* and *om*/*om* animals. In normal control +/*om* mice, all teeth, including molars, were present and erupted (Fig. 3a, data not shown). In *om*/*om* mice, we found that incisors had erupted but appeared damaged (data not shown). We also identified molars, but these were still covered with bone and an epithelial cell layer (Fig. 3b). This confirmed that molars failed to erupt in *om*/*om* mice.

To determine the onset of the incisor defect, we examined pups from +/*om* × *om*/*om* matings at several time points. Serial sections of teeth at E16.5 (*n* = 13) showed no obvious abnormalities in any of the embryos (data not shown). We observed 26 offspring from *om/om* × +/*om* matings from P14 until P30–32 and recorded the status of their incisors daily (Table 3). At the end of the time course, 13 mice were scored as affected (*om/om*) and 13 as normal (+/*om*) littermates. At P14, five mice, later identified as *om/om*, had apparently normal incisors. However, six *om/om* pups had small or thinner teeth and two mice had missing teeth. At P21, 2 of 13 *om/om* mice were scored as having normal incisors, two mice had small or thin teeth, and the remaining nine all had missing teeth. At P30–32, this trend continued and 11 mice had some teeth missing and two had no teeth visible at all. This indicated that the incisors were formed but not maintained.

**Table 3:** Number of control and *om*/*om* mice displaying a feature at P14, P21, and P30–32

	Teeth	P14	P21	P30–32
+/*om* (*n* = 13)	Normal size of teeth	13	13	13
	Normal number of teeth	13	13	13
*om/om* (*n* = 13)	Normal size of teeth	5	2	0
	Small/thin teeth	6	2	0
	Missing teeth^a^	2	9	11
	No teeth	0	0	2

All control mice had normal teeth at all time points. Incisors erupted in most *om*/*om* mice but degenerated after eruption

^a^Either top or bottom had teeth missing. Of these, six animals had two lower incisors, four animals had one lower incisor, one animal had one top and one lower incisor, and none had only top incisors

### *om*/*om* mice have an abnormal bone morphology and increased bone mass

Analysis of the skeletal structure by X ray showed that *om*/*om* mice had an abnormal skull shape as a result of malformed/eroded teeth. In the maxillary area the bone around the roots of the damaged upper incisors appeared rough. This sclerosis in the maxillary area appeared to get progressively worse, with a denser appearance at the 12-week time point (Fig. 2a–d, data not shown). The zygomatic process of the maxilla was thicker and continued to be thick halfway into the zygomatic bone (Fig. 2c, d). Proximal ribs 1–8 were thickened, especially on the dorsal parts (data not shown). Long bones had a layered appearance and there was an increased density of the deltoid tuberosity of the humeri (Fig. 3e, f) and of the diaphyses of the femurs in females (Fig. 2g, h). In addition, the deltoid process of the humerus had an abnormal morphology (Fig. 3e, f), and femurs were shorter but thicker and had increased density at the distal epiphysis. DEXA scan revealed that *om/om* mice had a significantly reduced lean and fat mass (Table 4). At 8 weeks, *om*/*om* females had a low bone mineral density (BMD), measured over the full body or the femur, when compared to +/*om* females (Table 4). At 12 weeks, the BMD of *om*/*om* females was significantly higher compared to that of +/*om* controls mice. BMD at the right knee of *om/om* females was higher at both time points compared to that of +/*om* females (Table 4). This suggests progressive osteopetrosis in *om/om* females. The BMD of *om*/*om* males was decreased at 8 weeks. At 12 weeks, the BMD of *om*/*om* males was similar to that of +/*om* males. This implies that the progressive osteopetrosis seen in young females is not present in the males at this age. Blood from +/*om* control and *om/om* mice was analysed. Blood counts were normal (Supplementary Table 3) but clinical chemistry at 16 weeks showed that *om*/*om* mice have high alkaline phosphatase levels (males: +/*om* = 68.24 U/l, *om/om* = 96.50 U/l; females: +/*om* = 77.40 U/l, *om/om* = 130.25 U/l, Supplementary Table 4). Although the level of total alkaline phosphatase (TAP) can be influenced by many factors, TAP is always raised in osteopetrosis patients and mouse models (Sanger et al. 1966; Carolino et al. 1998; Kornak et al. 2001). The higher alkaline phosphatase levels of *om/om* females may be consistent with the higher BMD seen at 12 weeks. Lower levels of triglycerides, LDL, and haemoglobin in homozygote mice are likely to be an indication of poor nutritional status in *om*/*om* mice but could be due to other underlying metabolic problems (Supplementary Table 4).

**Table 4:** Body mass and bone mineral densities (BMD) from control and *om*/*om* male and female mice at 8 and 12 weeks of age

		Lean mass (g)	Fat mass (g)	BMD (g/cm^2^)		
				Total body	Right knee	Right femur
Females	+/*om* (*n* = 7)	19.78	5.90	0.051	0.082	0.060
8 weeks	*om/om* (*n* = 4)	15.25	3.36	0.048	0.090	0.065
	*t* test	0.0027	0.0370	0.069	0.030	0.117
Males	+/*om* (*n* = 7)	23.95	6.74	0.053	0.088	0.066
8 weeks	*om/om* (*n* = 4)	18.07	3.95	0.387	0.082	0.064
	*t* test	1.17E–06	1.36E–02	0.033	0.025	0.756
Females	+/*om* (*n* = 7)	22.62	9.96	0.056	0.090	0.069
12 weeks	*om/om* (*n* = 4)	16.14	3.49	0.060	0.112	0.083
	*t* test	1.69E–05	5.55E–04	0.0014	0.0002	0.0013
Males	+/*om* (*n* = 7)	26.45	10.03	0.0548	0.09	0.07
12 weeks	*om/om* (*n* = 4)	19.53	5.87	4.431	0.086	0.066
	*t* test	1.59E–06	5.27E–05	0.506	0.828	0.028

We analysed haematoxylin and eosin-stained sagittal sections of the heads from 4-week-old +/*om* and *om*/*om* mice for tooth and bone abnormalities. In control mice molars had erupted, but in *om/om* mice molars were still covered by tissue (Fig. 3a, b). The jaw bones of control mice consisted of spongy bone and compacted bone areas (Fig. 3c, e). In the compacted bone, no blood vessels were seen and the bone had a regular structure (Fig. 3c). In contrast, the areas normally formed by compact bone were invaded by blood vessels and had a very irregular structure in *om*/*om* mice (Fig. 3d). In addition, the spongy bone appeared disorganised (Fig. 3f).

### The *omi* mutation maps to chromosome 10

To map the *omi* mutation, we crossed *om*/*om* mice with C57BL/6J mice. All offspring (*n* = 43) were normal (+/*om*) (Table 5). These outcrossed mice were then backcrossed to an affected *om*/*om* mouse. A total of 182 backcross mice were collected; 97 (53.3 %) had the tooth defect and a grey appearance (*om*/*om*) and 85 (46.7 %) were normal in appearance (+/*om*) (Table 5). These crosses indicate an autosomal recessive inheritance. DNA from 28 backcross offspring that had the tooth defect and a grey appearance was used to identify chromosome/trait linkage. Analysis of 69 polymorphic markers (Supplementary Table 1) distributed throughout the autosomes indicated clear linkage of the *omi* phenotype to chromosome 10 (data not shown). The highest percentage (92 %) of homozygosity for the C3H-type polymorphism was found at marker *D10Mit106*. To narrow down the region, we used additional markers on chromosome 10 of the same 28 backcross animals (Supplementary Table 2; data not shown). This narrowed the interval to the region of chromosome 10 between *D10Mit214* and *D10Mit194*, with the highest percentage of homozygosity at *D10Mit3* (100 %).

**Table 5:** Number of affected and normal mice in the out- and backcross confirms recessive inheritance

	Outcross		Backcross	
	(*om/om* × C57Bl6 J)		(outcross × *om/om*)	
Offspring	*n* = 43	(%)	*n* = 182	(%)
*om/om*	0	0	97	53.3
+/*om*	43	100	85	46.7

**Fig. 4:**
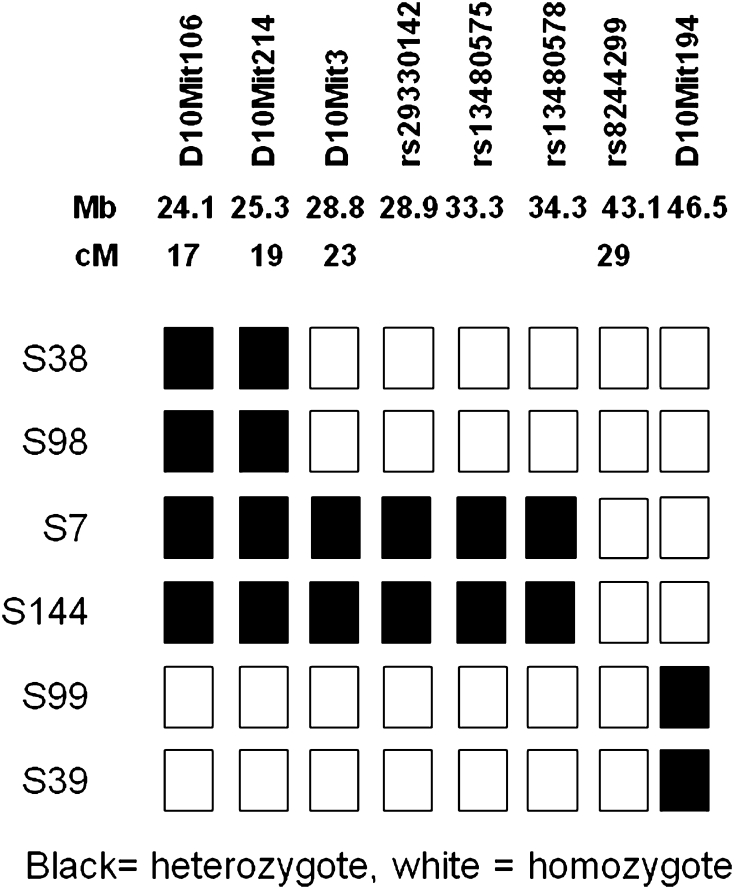
Genetic mapping of the *omi* mutation. A genome-wide association scan using Mit markers located the mutation on chromosome 10. Fine mapping using Mit markers and SNPs polymorphic between C3H and C57 mice showed that the mutation lies between rs13480578 and *D10Mit194*. *S(number)* sample name, *black* sample is heterozygous for a marker, *white* sample is homozygous for a marker, *Mb* position of marker on chromosome 10 in megabases

An additional set of 44 *om*/*om* backcross samples was used to confirm the data and to narrow the genomic interval. The haplotypes of the animals defining this critical region are shown in Fig. 3b. One animal was heterozygote for *D10Mit3* but homozygote for *D10Mit194*, narrowing the region to this interval. This corresponds to a 17.6-Mb physical region from 28.8 to 46.5 Mb on chromosome 10. We then used four SNPs polymorphic between C57 and C3H that are located in the region between *D10Mit3* and *D10Mit194* (Fig. 4). The six animals that set the critical interval were used (Fig. 4). Two animals (S7 and S144) were heterozygote for C3H and C57 alleles at SNPs rs29330142, rs13480575, and rs13480578. All animals were homozygous for the C3H allele of SNP rs8244299 at 43.0 Mb. Therefore the *omi* mutation lies in the 12-Mb region between SNP rs13480578 and *D10Mit194*.

### *Omi* and *grey-lethal* are noncomplementing mutations

**Fig. 5:**
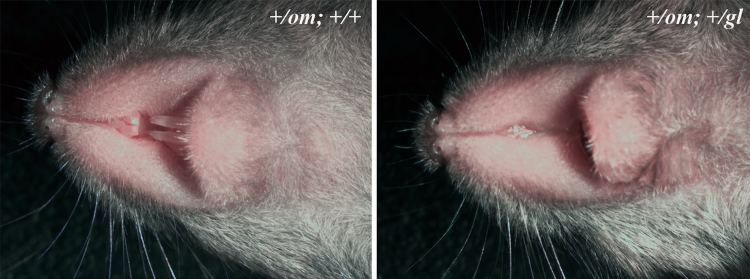
Genetic complementation. Homozygous *omi* (*om/om*) mice were crossed with heterozygous *grey-lethal* (+/*gl*) mice and offspring were analysed for gross morphological defects. **a** Three-week-old +/*om*; +/+ mice have normal teeth, **b** A 3-week-old littermate (+/*om*; +/*gl*) has missing incisors and coat colour abnormalities. Unlike *gl*/*gl* mice, these compound heterozygotes are viable until at least 12 weeks of age

The region on chromosome 10 between SNP rs13480578 and *D10Mit194* contains approximately 100 genes. Several genes in the region could lead to a severe phenotype as seen in the *om*/*om* mice, but one gene was of particular interest. *Ostm1* mutations have been identified in mouse and human as causing very severe osteopetrosis. *Grey-lethal* (*gl*) mice have a recessive null mutation in *Ostm1* that leads to a grey coat colour and early lethality due to severe osteopetrosis (Chalhoub et al. 2003; Pangrazio et al. 2006). Due to the similarity in phenotype between the *grey-lethal* and *omi* phenotypes, we tested for noncomplementation. Offspring of crosses between *om*/*om* and +/*gl* mice were collected. Of 28 pups, 10 had a grey appearance and lacked incisors at P21 and the remaining pups were all normal (Fig. 5a, b). These ten abnormal pups were confirmed to be heterozygous for the *grey-lethal* allele by genotyping (and thus +/*gl*, +/*om*). In contrast to *gl*/*gl* mice, that are small at P21 and lethal around 3–4 weeks of age, these compound heterozygous mice were viable on a Pico-Vac^®^ soft diet for at least 12 weeks.

### *om*/*om* mice do not have a mutation in the coding sequence and 500-bp promoter region of *Ostm1* but have reduced Ostm1 protein levels

We sequenced the coding region of *Ostm1* in *om/om,* +/*om*, and control mice from wild-type C3HeB/FeJ colony (+/+) mice. We were unable to detect a mutation within the coding sequence or the 5′ and 3′ UTRs. Work by Meadows et al. (2007) has shown that *Ostm1* expression is regulated by the MIcrophthalmia Transcription Factor (MITF). Analysis of the alignment of human and mouse areas upstream (500 bp) of the transcription start site identified one putative MITF binding site (M box) and three weaker binding sites (E boxes). In addition, Ets and Pu.1 binding sites were identified (Meadows et al. 2007). We sequenced this region for control and *om*/*om* mice and did not find any mutation in this promoter region.

**Fig. 6:**
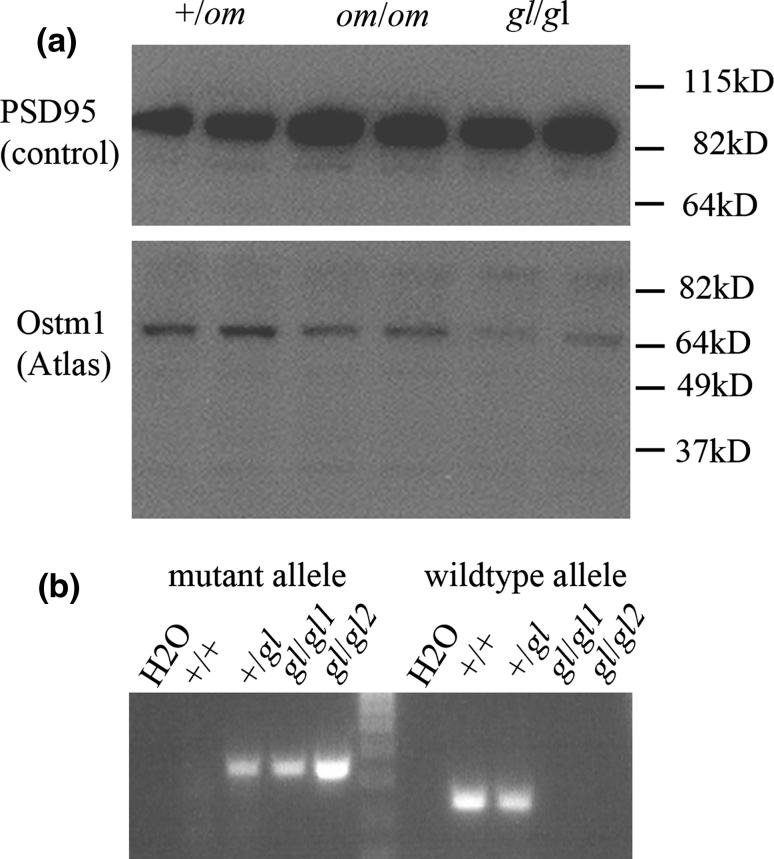
Ostm1 protein levels in +/*om*, *om*/*om*, and *gl/gl* mice analysed by Western blotting. **a** Brain lysates of 3-week-old +/*om*, *om*/*om*, and *gl/gl* mice were analysed by Western blotting for the Ostm1 protein. Blots were stripped and incubated with a PSD95 antibody to determine sample loading, **b** Genotypes of *grey-lethal* (*gl/gl*) samples were verified by PCR analysis

Next we analysed Ostm1 protein levels of the brains of +/*om*, *om*/*om*, and *gl*/*gl* mice. The OSTM1 antibody recognized several bands ranging from 35 to 90 kDa, with the strongest band just above 64 kDa. This band was weaker in *om/om* mice and weakest in *gl/gl* mice compared to the loading control (Figs. 5, 6). This suggests that *om/om* mice have less Ostm1 protein than normal mice but more than *gl/gl* mice.

## Discussion

Here we described a novel allele (*omi*) of *Ostm1* that results in tooth, bone, and coat colour abnormalities. All homozygote mutant (*om*/*om*) mice lose incisors in the first month of life and most molars fail to erupt. We have demonstrated that female *om/om* mice may suffer from progressive osteopetrosis at a young age. Male *om*/*om* mice have decreased BMD at 8 weeks of age, but bone mass increases significantly between 8 and 12 weeks of age, even though mice suffer from poor nutritional status (indicated by low triglycerides, LDL, reduced lean and fat mass). In addition, *om/om* mice have a shortened life span. It is possible that bone mass would increase further with age in *om*/*om* males and an osteopetrotic phenotype could underlie the shortened life span, but further detailed analysis of this complex bone phenotype is required.

The *omi* mutation is likely to have been induced by ENU mutagenesis. A spontaneous mutation rate of 1.1 × 10^−8^ per base per generation was estimated for the mouse (Drake et al. 1998), resulting in an average of 28.6 mutations per genome per generation. In contrast, the mutation rate induced by ENU can be approximately 10,000 times higher than the natural background mutation rate (Salinger and Justice 2008; http://cshprotocols.cshlp.org/content/2008/4/pdb.prot4985.full), for an average of 3.97 × 10^7^ mutations in the first generation. After five generations of outcrossing with unexposed C3HeB/FeJ, one would expect 93.75 % of the mutations to have been diluted out, leaving around 2.48 × 10^5^ ENU-induced mutations. Therefore, it is likely that the mutation was ENU-induced, but we cannot exclude the possibility that it arose spontaneously.

The phenotype of the *om/om* mice is similar but not identical to the previously reported *grey-lethal* mouse. *Grey-lethal* is a recessive mutation in *Ostm1* but has a more severe phenotype than the *om*/*om* phenotype across all aspects of the phenotype. Incisors and molars do not erupt in *grey-lethal* mice. Some teeth do erupt in *om/om* mice but most are not maintained and are lost just after weaning. In vitro cultures of tooth germs showed that teeth of *grey-lethal* mice can develop normally, and the failure to erupt in vivo is due to an absence of bone remodelling (Ida-Yonemochi and Saku 2002). *Grey-lethal* mice are not viable and die around weaning time, even when the mice are on an appropriate soft diet, whereas *omi* mice are viable and fertile on a soft gel diet. The grey coat colour is another phenotype that is more severely affected in *grey-lethal* mice than in *omi* mice. The *grey-lethal* mutation has been bred onto various genetic backgrounds, including C3H and C57BL/6J. The phenotype of the *grey-lethal* mouse is less severe on a *Mus spretus* genetic background, indicating that modifier genes exist (Vacher and Bernard 1999). The *omi* mutation arose on an inbred C3HeB/FeJ genetic background and backcrossing to C57BL/6J did not alter the phenotype significantly. *Grey-lethal* was discovered by Grüneberg (1935) in a stock segregating for *Tyr* ^*c*–*e*^. It arose as a spontaneous mutation in a mixed genetic background. Based on mapping data, Vacher and Bernard (1999) suggested that the mutation arose on a 129 or related genetic background. The *grey-lethal* mutation was identified as a genomic deletion of the 5′ region of the gene. The deletion spans 7.5 kb and includes the promoter, the first exon, and part of the first intron. A 460-bp sequence corresponding to the 3′ UTR of a LINE1 retrotransposon element was inserted at the deletion breakpoints (Chalhoub et al. 2003). RT-PCR showed that this *Ostm1* mRNA is not expressed in *gl*/*gl* tissues, making it likely that this is a complete null mutation.

One possibility is that the difference in the type of mutation could cause the difference in phenotype. *Omi* was identified in a mouse line that came from an ENU mutagenesis program. Therefore, it is likely to be a point mutation rather than a retroviral integration as is the *grey-lethal* mutation. *Omi* could be a hypomorphic allele rather than a complete null mutation, which would explain the difference in phenotype. Our data suggest that *om/om* mice have reduced levels of Ostm1 protein, confirming that it is not a null mutation. Our current hypothesis is that the *omi* mutation could lie in a regulatory element and lead to a reduction in *Ostm1* expression. As enhancer elements have been identified at many kilobases up- or downstream from the transcription start site, further fine mapping of the *omi* mutation in combination with new sequencing technologies will be required to identify the causative mutation.

Osteopetrosis in humans was described in the early 1900s by Albers-Schönberg, and by the 1940s it was clear that the severity of the phenotype varied greatly. In the most severe forms, deficient bone resorption leads to haematological failure, cranial nerve compression, short stature, and brittle bones. Various inheritance patterns have been described for the different types of osteopetrosis. Autosomal dominant osteopetrosis is variable but usually mild. Various forms of recessive osteopetrosis have been reported, some of which are associated with the most severe phenotypes (including those caused by mutations in the *TCIRG1*, *CLC7*, and *OSTM1* genes), whereas mutations in other genes (*CAII* and *PLEKHM1*) give a milder osteopetrotic phenotype. Recently, long-term survival in infantile malignant autosomal recessive osteopetrosis secondary to homozygous p.Arg526Gln mutation in *CLCN7* was described (Kantaputra et al. 2012). Interestingly, the phenotype of *omi* mice greatly overlaps the description of a case study of recessive mild osteopetrosis (Kahler et al. 1984). As in *omi* mice, patients presented with oligodontia, striation of the long bones, abnormalities of the zygomatic arch, sclerosis in the skull, and mild anaemia. To date, only four different human *OSTM1* mutations have been identified, always leading to recessive malignant osteopetrosis. This does not exclude the possibility that other hypomorphic *OSTM1* mutations could lead to a mild osteopetrotic phenotype in humans. The recent identification of a similar mutation in *CLC7* (Kantaputra et al. 2012) confirms the idea that many more subtle mutations may be identified in the human population. Therapy for osteopetrosis is presently unsatisfactory and much work needs to done to unravel the gene defects and to identify new treatments to improve symptoms. Recent efforts to identify novel genes involved in bone homeostasis by using ENU mutagenesis screening in mice have identified one novel candidate for osteopenia (Barbaric et al. 2008) and one for osteopetrosis (Ochotny et al. 2011). Although *omi* mice have a mutation that affects a gene already known to be involved in bone homeostasis, it is likely to be hypomorphic making it a suitable model for validating novel therapeutic treatments.

## Electronic supplementary material

Below is the link to the electronic supplementary material.

Supplementary material 1 (PDF 40 kb)
