# Supplementary material for: Omi, a recessive mutation on chromosome 10, is a novel allele of Ostm1
Source: Mamm Genome. 2012 Nov 17;24(1):44–53. doi: 10.1007/s00335-012-9438-7 (PMC3560959; doi:10.1007/s00335-012-9438-7)
Supplement: Supplementary file 1 — Supplementary material 1 (PDF 40 kb) [file 335_2012_9438_MOESM1_ESM.pdf]

Supl Table 1

marker	cM
D1Mit429	8.4
D1Mit156	32.8
D1Mit21	33.89
D1Mit415	53.06
D1Mit353	90.34
D2Mit369	27.3
D2Mit237	28
D2Mit107	75.6
D2Mit59	86
D2Mit200	98.36
D3Mit117	2.35
D3Mit339	29.25
D3Mit199	56.1
D3Mit258	70.3
D3Mit19	87.6
D4Mit172	11.3
D4Mit111	21.9
D4Mit58	40.62
D4Mit203	60
D4Mit33	67.6
D5Mit345	1
D5Mit168	78
D5Mit292	80
D6Mit159	7
D6Mit268	15.6
D6Mit243	30.4
D6Mit366	43.69
D6Mit254	60.55
D7Mit178	0.5
D7Mit230	22.45
D7Mit66	57.5
D8Mit339	23
D8Mit113	52
D8Mit321	59
D8Mit280	72
D9Mit224	17
D9Mit162	30
D9Mit214	58.79
D10Mit106	17
D10Mit115	38.4
D10Mit12	56
D10Mit180	65.22
D10Mit271	70
D11Mit140	25.73
D11Mit35	44.74
D11Mit99	63.21
D12Mit69	23.88
D12Nds2	59
D13Mit3	8.96
D13Mit88	21
D14Mit260	21.21
D14Mit225	44.1
D14Mit266	60
D15Mit175	5.72

marker	cM
D15Mit43	58.01
D16Mit165	11.17
D16Mit63	
D16Mit152	48
D16Mit106	71
D17Mit238	2.22
D17Mit126	21.8
D17Mit155	55.7
D18Mit64	2
D18Mit37	21
D18Mit187	32.54
D18Mit40	41
D19Mit59	0
D19Mit16	26
D19Mit137	55.7

For Peer Review

Supl Table 2

marker	Bp (ensembl)	
D10Mit206	13796038	
D10Mit168	18496132	
D10Mit212	18320778	
D10Mit51	unknown	
D10Mit213	20096855	
D10Mit106	24079119	
D10Mit214	25338521	
D10Mit3	28841602	
D10Mit194	46532722	
D10Mit115	69672053	
D10Mit12	98903244	
D10Mit180	117553604	
D10Mit271	123749006	
Timeless	127635041	
rs8244299	43075748	SNP1
rs13480575	33342439	SNP3
rs29330142	28870557	SNP4
rs13480578	34267269	SNP8

For Peer Review

Supl Table 3

Gender	Genotype	Age		MCH	MCV	PLT	HCT	HGB
female	+/*om*	16 weeks	Average	15.3025	56.75	1240	51.7925	13.96
			std	0.308153	0.957427	46.71902	1.300702	0.391067
female	*om* /*om*	16 weeks	Average	15.085	56.5	1083	53.645	14.325
			std	0.120208	0.707107	82.02439	0.473762	0.205061
male	+/*om*	16 weeks	Average	15.0025	55.5	1095.25	53.94	14.5675
			std	0.207103	0.57735	100.6028	1.240672	0.386986
male	*om* /*om*	16 weeks	Average	14.745	55.5	1047.5	52.94	13.965
			std	0.289914	0.707107	6.363961	0.46669	0.205061

Gender	Genotype	Age		RBC	WBC	MCHC	RDW	MPV
female	+/*om*	16 weeks	Average	9.1275	5.8725	26.9575	12.405	5.8875
			std	0.233006	0.802304	0.332904	0.395685	0.205163
female	*om* /*om*	16 weeks	Average	9.5	4.605	26.705	11.63	5.69
			std	0.212132	0.997021	0.148492	0.056569	0.183848
male	+/*om*	16 weeks	Average	9.715	6.3725	27.005	11.8875	6.1225
			std	0.30304	0.812091	0.280535	0.406479	0.282887
male	*om* /*om*	16 weeks	Average	9.475	6.86	26.38	11.935	6.275
			std	0.049497	0.254558	0.141421	0.13435	0.388909

For Peer Review

Supl Table 4

Gender	Genotype	Age		NA	K	CL	GLUC	TRIGS
female	+/*om*	16 weeks	Average	141.375	5.3	104.075	31.975	3.5875
			std	1.087428	0.08165	0.5058	2.528906	0.333504
female	*om*/*om*	16 weeks	Average	145.45	4.5	108.9	15.465	1.195
			std	1.484924	0.424264	0.424264	3.627458	0.671751
male	+/*om*	16 weeks	Average	146.85	5.2	107.525	23.7575	4.815
			std	2.46103	0.355903	0.991211	1.439198	1.067474
male	*om*/*om*	16 weeks	Average	149.4	5.6	109.8	18.735	1.315
			std	0.282843	0.282843	0.989949	1.902117	0.388909

For Peer Review

Supl Table 4 cont.

Gender	Genotype	Age		CHOL	AMY	NEFAC	TP	ALB
female	+/*om*	16 weeks	Average	4.01	557.5	0.45	51.275	26.6725
			std	0.204124	27.59928	0.1	0.607591	1.075465
female	*om*/*om*	16 weeks	Average	3.045	445.4	0.3	51.4	27.445
			std	0.205061	36.48671	0	3.535534	2.170818
male	+/*om*	16 weeks	Average	4.595	657.05	0.9	51.825	25.1875
			std	0.377757	40.43451	0.182574	1.228481	0.898271
male	*om*/*om*	16 weeks	Average	4.25	455.7	0.65	53.7	25.565
			std	0.39598	39.03229	0.070711	0.707107	0.346482

For Peer Review

Supl Table 4 cont.

Gender	Genotype	Age		CA	PHOS	IRON	AST	ALP
female	+/*om*	16 weeks	Average	2.3625	3.48	33.5	50.125	79.875
			std	0.020616	0.156631	3.569314	4.04753	9.492585
female	*om*/*om*	16 weeks	Average	2.43	3.08	30.15	57.15	130.25
			std	0.014142	0.028284	6.858936	3.464823	1.343503
male	+/*om*	16 weeks	Average	2.3275	3.2025	32.35	45.425	68.25
			std	0.101448	0.283593	2.275229	8.328815	3.250128
male	*om*/*om*	16 weeks	Average	2.385	3.4	31.85	57.1	96.5
			std	0.035355	0.353553	2.757716	4.525483	0.848528

For Peer Review

Supl Table 4 cont.

Gender	Genotype	Age		ALT	CREAT	LDH	UREA	TBILC
female	+/*om*	16 weeks	Average	48.5	15	328.25	9.7	2.275
			std	3.906405	4.786091	75.40029	0.69282	0.35
female	*om*/*om*	16 weeks	Average	21.8	14.8	412.3	8.45	2.05
			std	0.141421	3.252691	41.01219	3.747666	0.070711
male	+/*om*	16 weeks	Average	35.85	12.475	369.25	9.325	2.325
			std	4.939973	2.245551	67.25774	1.212092	0.320156
male	*om*/*om*	16 weeks	Average	44.85	10.95	504.3	7.35	2.4
			std	3.181981	1.202082	28.14285	1.06066	0.424264

For Peer Review

Supl Table 4 cont.

Gender	Genotype	Age		CK	MG	HDL	LDL	UA
female	+/*om*	16 weeks	Average	122.75	0.9925	2.8625	0.4425	10.125
			std	43.24392	0.061305	0.14975	0.027538	2.960152
female	*om*/*om*	16 weeks	Average	120.15	0.925	2.18	0.44	21.6
			std	14.91995	0.049497	0.084853	0.028284	3.394113
male	+/*om*	16 weeks	Average	149.925	0.935	3.25	0.5875	9
			std	97.2978	0.067577	0.228473	0.025	2.736177
male	*om*/*om*	16 weeks	Average	199.95	0.83	3.27	0.525	32.3
			std	33.30473	0.070711	0.240416	0.091924	0.707107

For Peer Review
